# Colloidal Silver Nanoparticles Obtained via Radiolysis: Synthesis Optimization and Antibacterial Properties

**DOI:** 10.3390/pharmaceutics15071787

**Published:** 2023-06-21

**Authors:** Mario Menéndez Miranda, Wenbo Liu, Jesus Alfredo Godinez-Leon, Aisara Amanova, Ludivine Houel-Renault, Isabelle Lampre, Hynd Remita, Ruxandra Gref

**Affiliations:** 1 Institut de Sciences Moléculaires d’Orsay, CNRS-UMR 8214, Université Paris-Saclay, 91400 Orsay, France; mmiranda@serida.org (M.M.M.); wenbo.liu@universite-paris-saclay.fr (W.L.); jesus.godinez@universite-paris-saclay.fr (J.A.G.-L.); ludivine.houel-renault@universite-paris-saclay.fr (L.H.-R.); 2 Institut de Chimie Physique, CNRS-UMR 8000, Université Paris-Saclay, 91405 Orsay, France; aisara.amanova@universite-paris-saclay.fr (A.A.); isabelle.lampre@universite-paris-saclay.fr (I.L.)

**Keywords:** silver nanoparticles, *γ*-irradiation, radiolysis, antibacterial property, *S. aureus*, sterile colloidal suspensions, bactericidal ability

## Abstract

Silver nanoparticles (AgNPs) with broad-spectrum antimicrobial properties are gaining increasing interest in fighting multidrug-resistant bacteria. Herein, we describe the synthesis of AgNPs, stabilized by polyvinyl alcohol (PVA), with high purity and homogeneous sizes, using radiolysis. Solvated electrons and reducing radicals are induced from solvent radiolysis and no other chemical reducing agents are needed to reduce the metal ions. Another advantage of this method is that it leads to sterile colloidal suspensions, which can be directly used for medical applications. We systematically investigated the effect of the silver salt precursor on the optical properties, particle size, and morphology of the resulting colloidal AgNPs. With Ag_2_SO_4_ precursor, the AgNPs displayed a narrow size distribution (20 ± 2 nm). In contrast, AgNO_3_ and AgClO_4_ precursors lead to inhomogeneous AgNPs of various shapes. Moreover, the optimized AgNPs synthesized from Ag_2_SO_4_ were stable upon storage in water and phosphate-buffered saline (PBS) and were very effective in inhibiting the growth of *Staphylococcus aureus* (*S. aureus*) at a concentration of 0.6 μg·mL^−1^ while completely eradicating it at a concentration of 5.6 μg·mL^−1^. When compared with other AgNPs prepared by other strategies, the remarkable bactericidal ability against *S. aureus* of the AgNPs produced here opens up new perspectives for further applications in medicine, cosmetics, the food industry, or in elaborating antibacterial surfaces and other devices.

## 1. Introduction

Metallic silver, which is the third precious metal known by ancient civilizations after gold and copper, is often used in daily life and in industrial areas, in electronics, jewelry, and medicine [1,2,3]. Until the 1940s, silver found many applications to treat wounds and infections, as well as other diseases [4]. Then, the development of antibiotics like penicillin gradually supplanted the use of silver-based formulations [5]. However, the widespread use of antibiotics led to the emergence of bacterial resistance, which in turn led to the revival of the use of silver-based medicines.

**Figure 1:**
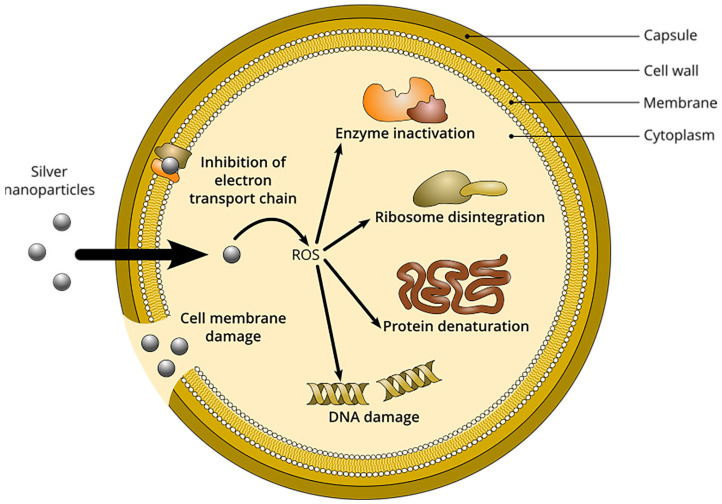
Possible pathways of action of silver nanoparticles against *S. aureus*, involving reactive oxygen species (ROS) production, cell membrane and DNA damages, ribosome disintegration, protein denaturation, inhibition of electron transport, as well as enzyme inactivation.

In particular, silver nanoparticles (AgNPs) have garnered significant research interest in recent years [2,3,6,7]. AgNPs are defined as clusters of silver atoms in the size range 1–100 nm with various shapes [8]. In contrast to their corresponding bulk counterparts, nanomaterials have distinct chemical and physical properties due to their large surface area and size confinement effects. Indeed, the physicochemical and antimicrobial properties of AgNPs and other silver-based devices such as films or fabrics are closely associated with their size, shape, and stability, which, in turn, depend on the preparation method and formulation parameters [9,10]. Numerous reports highlight the antibacterial properties of AgNPs schematically illustrated in Figure 1. These nanoscale particles can penetrate bacterial cell membranes, increasing the cell permeability or damaging the bacterial cell wall [11]. Additionally, the release of bioactive Ag^+^ from colloidal AgNPs can produce reactive oxygen species (ROS), which can interfere with metabolic pathways, inhibit DNA replication, affect the synthesis of enzymes and proteins, and disintegrate ribosomes [12]. Versatile AgNP materials are produced through different syntheticprotocols. The chemical reduction method is frequently used in the process of creating nano-silver [13,14]. Nevertheless, this method relies on the use of potentially dangerous reducing agents such as sodium borohydride or hydrazine hydrate. Therefore, researchers are gradually focusing on new promising synthesis approaches including photochemical method [15,16,17], microwave-assisted synthesis [18], “green” synthesis based on plant or microorganism extracts, [19] and radiolysis methods [20,21,22].

Among these strategies, radiolysis is a powerful method allowing synthesis of nanoparticles with controlled size and shape both in solution and in heterogeneous media [22,23]. Reducing species (solvated electron and radicals) are induced by solvent radiolysis. Therefore, no chemical reducing agents are needed to reduce the metal ions, which limits the contamination of the synthesis medium and avoids further purification steps. Radiolysis induces very homogeneous reduction and nucleation. Furthermore, the irradiation leads to sterilization of the metal NP suspensions, which is very convenient and a major advantage for medical applications. The groups of Belloni and Henglein were the first to report on the radiolytic synthesis of AgNPs and to study the reduction and growth mechanisms [20,24,25,26,27].

Usually, colloidal AgNPs are stabilized to prevent their agglomeration using a variety of ligands, polymers, or surfactants. Sheikh and co-workers reported colloidal AgNPs obtained with silver nitrate as metal precursor and polyvinylpyrrolidone (PVP) polymer as stabilizer, using *γ*-irradiation [28]. Du et al. also developed colloidal AgNPs by using AgNO_3_ in PVP solution though *γ*-irradiation and investigated the effect of PVP molecular weight and ionic concentration of AgNO_3_ on silver particles size [29]. Additionally, various stabilizers, like *β*-D-glucose, chitosan, gelatin, carboxymethyl cellulose, calixarenes, and polyvinyl alcohol (PVA) [18,27,30,31,32], have also been successively employed in the preparation of nanoparticles by radiolysis. Recently, Cui et al. synthesized poly(3,4-ethylenedioxythiophene) (PEDOT)-stabilized Ag nanocomposites from silver perchlorate as the silver source by radiation-assisted synthesis and systematically compared two synthesis strategies (two-step method and one-pot method) via optimized reaction parameters involved in radiation [33]. In addition, Duteanu et al. reported a new generation of antibacterial products based on colloidal silver, which were derived from soluble starch used as green reducing agent and stabilizer, and studied the influence of the reducing agent on the size of AgNPs [34].

However, only a few reports in the literature have dealt with antibacterial or antifungal properties of radio-synthesized AgNPs. Mahmoud et al. prepared a nanocomposite hydrogel containing AgNPs using PVA as a stabilizer and crosslinker [35]. The hydrogel inhibited the growth of Gram-positive and Gram-negative bacteria, as well as the fungi *Candida albicans.* In other studies, Qi et al. developed an antibacterial fabric with anchored AgNPs through *γ*-irradiation-induced graft reduction. This fabric was endowed with both a good mechanical strength and an excellent antibacterial ability against *Staphylococcus aureus* (*S. aureus*) (bacterial lethality >99%) [36]. Inhibition of fungal growth was observed using a TiO_2_ surface modified with AgNPs and small Cu clusters synthesized by radiolysis [22].

Likewise, silver nitrate has been widely employed to obtain versatile AgNPs, both with traditional chemical methods and in other synthetic strategies including radiosynthesis [31,32,35,37,38]. However, few studies have been conducted on the influence of the type of silver salt precursor on the physical and chemical properties of the obtained colloidal AgNPs.

Therefore, there is a clear need to investigate systematically the use of various precursors in the synthesis of AgNPs. In this study, taking advantage of the radiolytic synthesis producing sterilized AgNP suspensions, ready for use in biological applications, AgNPs were obtained using three different silver salts (silver nitrate, silver sulfate, silver perchlorate) and PVA as stabilizer. PVA is a water-soluble and non-toxic polymer extensively used in the biomedical field [39]. The influence of the concentration of silver salt and the radiation dose on the optical properties of AgNPs were also studied in detail. The stability in water and phosphate-buffered saline (PBS) solutions for the as-prepared AgNPs was investigated. Finally, the antibacterial properties of the AgNPs against *S. aureus* were evaluated, and higher antibacterial activity than those already reported for AgNPs synthesized by other methods was found.

## 2. Materials and Methods

### 2.1. Materials

All chemicals were of reagent grade purity and were used without further purification. Silver sulfate (Ag_2_SO_4_), silver perchlorate (AgClO_4_), silver nitrate (AgNO_3_), 2-propanol ((CH_3_)_2_CHOH), polyvinyl alcohol (PVA, 87–90% hydrolyzed, MW 30,000–70,000 g/mole), potassium chloride (KCl, ≥99%) and sodium hydroxide (NaOH, ≥99.0%) were obtained from Sigma Aldrich (Saint Quentin Fallavier, France). Luria-Bertani (LB) broth powder and agar powder for the preparation of bacterial culture media were purchased from Becton, Dickinson and Co., France. *S. aureus* ATCC 27,217 bacteria strain was obtained from the American type culture collection (ATCC). The water used for the preparation of the solutions was purified using a Millipore MilliQ system at 18.2 MΩ·cm at 25 °C.

### 2.2. Preparation of AgNPs

The radiolytic synthesis of AgNPs were performed as described elsewhere [21,40], using a panoramic *γ*-irradiation source with a ^60^Co γ-facility at a maximum dose rate of 1.18 Gy·s^−1^ (4250 Gy·h^−1^) at Institut de Chimie Physique, Orsay, France. Typically, PVA powder and different silver salts (Ag_2_SO_4_, AgClO_4_, and AgNO_3_) were added to deionized water, and then stirred to form mother solutions, respectively. For dissolution of PVA, the mother solutions were heated and stirred at 100 °C for one hour until the solution became completely transparent. Mixtures, containing silver ions (1 and 2 mM) from distinct silver salts as precursor, PVA (100 mM) as stabilizer, and 2-propanol (100 mM) as oxidizing radical scavenger, were bubbled with nitrogen gas (99.5%, from Air Liquide), and then exposed to radiation with different irradiation doses at room temperature. After irradiation, the nanoparticle suspensions were stored in the dark to avoid light damage.

### 2.3. Characterization

UV-visible absorption spectra were recorded with a single beam Hewlett-Packard 8453 spectrophotometer. Twenty-five µL of the irradiated solutions were diluted in 1 mL water in a 1cm optical path cuvette. Transmission electron microscopy (TEM) observations were performed with a JEOL 100CXII TEM at an accelerating voltage of 100 kV. Samples for TEM analysis were prepared by placing drops of AgNP suspensions on carbon coated copper grids for TEM observations. The size distribution was determined using the ImageJ software based on the observation of at least 100 nanoparticles.

A Zetasizer (nano ZS series, Malvern Instruments Ltd., Malvern, UK) was used to evaluate the hydrodynamic diameter (HD, nm) and zeta potential (ZP, mV) of colloidal AgNPs, which were obtained via dynamic light scattering (DLS) and laser Doppler velocimetry (LDV), respectively. The pH of the colloidal AgNP solutions was adjusted to a pH of 7 at 25 °C using 0.1 M NaOH solution, without or with 0.01 M KCl solution for particle size measurement and surface charge, respectively.

### 2.4. In Vitro Evaluation of Antibacterial Ability

The Gram-positive bacterial strain of *S. aureus* ATCC 27,217 was incubated with gentle shaking at 120 RPM in sterile LB broth media at 37 °C overnight. The colony-forming units of *S. aureus* were determined using a Malassez counting chamber (Paul Marienfeld, Lauda-Königshofen, Germany), and adjusted to a bacterial concentration of 2 × 10^6^ CFU·mL^−1^ by diluting with sterile LB liquid. The antibacterial activity of radiation-synthesized AgNPs against *S. aureus* was assessed using the broth microdilution method. In brief, LB medium (100 µL) containing *S. aureus* at a concentration of 2 × 10^6^ CFU·mL^−1^ was added to a 96-well plate. A series of equal volumes of AgNP solution (100 µL) with different concentrations was added to each well.

The non-inoculated LB medium with AgNPs or different reaction components served as a negative control, while sterilized water and incubated bacteria were used as a positive control; the mixture of sterilized water and non-inoculated LB medium was defined as the blank control group. The 96-well plate was then incubated at 37 °C for 18–21 h. To determine the antibacterial activity of the synthesized AgNPs, the overnight-incubated bacteria mixture was serially diluted, then plated onto a culture agar plate, and incubated at 37 °C for 24 h. The number of bacterial colonies growing on the plate was counted visually to determine the CFU for each concentration, the minimum inhibitory concentration (MIC), and the minimum bactericidal concentration (MBC).

## 3. Results and Discussion

### 3.1. AgNP Synthesis and Optimization

**Figure 2:**
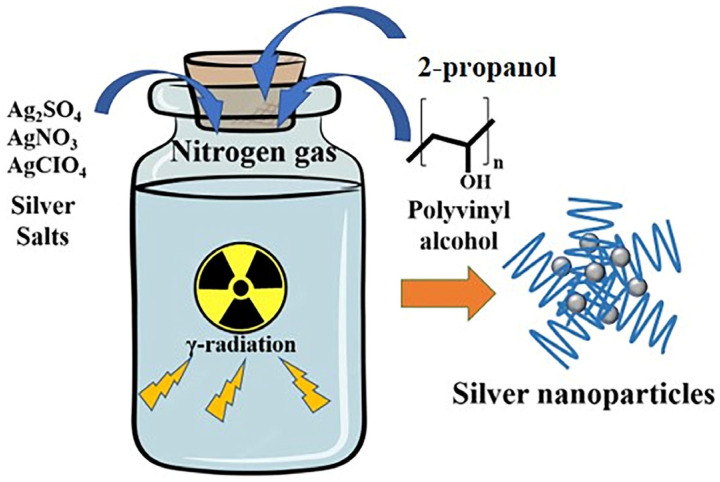
Schematic illustration of the radiation-assisted preparation of silver nanoparticles.

To compare the size distribution of Ag NPs obtained by radiolysis with different Ag precursors and study their antibacterial properties, we used here three different silver salts (Ag_2_SO_4_, AgNO_3,_ and AgClO_4_) at two different Ag^+^ concentrations, 1 mM and 2 mM. De-aerated aqueous solutions containing one of the three silver salts, 100 mM PVA as stabilizing agent, and 0.1 M 2-propanol (added as hydroxyl radical scavenger) were irradiated with γ-rays at different doses using a ^60^Co source at a dose rate of 4.25 kGy·h^−1^. The synthesis protocol is illustrated in Figure 2.

Briefly, the primary effects of irradiation by high-energy radiations (gamma rays, X-rays, electron beams, ion beams) are the ionization and excitation of the solvent water molecules generating reactive species such as hydrated electrons (e^−^_aq_), hydrogen radicals (H^•^), as well as hydroxyl radicals (OH^•^) (Reaction (1)).
H_2_O 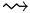 e^−^_aq_, H^•^, OH^•^, H_3_O^+^, H_2_O_2_, H_2_(1)

Hydrated electrons (e^−^_aq_), as powerful reducing agents (E°(H_2_O/e^−^_aq_) = −2.8 V_SHE_ [33,41]) are capable of reducing silver ions from the salt sources (Ag_2_SO_4_, AgNO_3,_ and AgClO_4_) to zero-valent silver atoms (Reaction (2)) [42].

Ag^+^ + e^−^_aq_ → Ag^0^ (2)

Hydrogen radicals (H^•^) and hydroxyl radicals (OH^•^) from irradiated water are free radicals with strong reducing and oxidizing properties, respectively, with redox potentials equal to E^0^(H^+^/H^•^) = −2.7 V_SHE_ and E^0^(HO^•^/H_2_O) = 2.3 V_SHE_ at pH value of 7 [43]. 2-propanol was employed as a radical scavenger of hydroxyl radicals, but it also reacts with H^•^ radicals, so, both HO^•^ and H^•^ radicals are converted into reducing alcohol radicals (Reactions (3) and (4)).

HO^•^ + (CH_3_)_2_CHOH → (CH_3_)_2_C^•^OH + H_2_O (3)

H^•^ + (CH_3_)_2_CHOH → (CH_3_)_2_C^•^OH + H_2_ (4)

The alcohol radicals have reducing properties and take part in the silver reduction, but their redox potential (E°((CH_3_)_2_CO, H^+^/(CH_3_)_2_C^•^OH) = −1.7 V [43]) is not sufficiently negative for a direct reduction of free silver ion to the atom (E°(Ag^+^/Ag^0^) = −1.8 V [44]). However, it has been shown that the reduction of free silver ions by alcohol radicals proceeds via the formation of a complex involving the metal ion and the alcohol radical, which acts as a ligand. This has been observed in the case of 2-propanol [44]. The alcohol radicals (and the hydrated electron) can also reduce silver ions on small radio-induced clusters as the redox potential of the metallic clusters Ag_n_^+^ increases with the nuclearity [45] (Reactions (5) and (6)).

Ag_n_^+^ + e^−^_aq_ → Ag_n_ (5)

Ag_n_^+^ + (CH_3_)_2_C^•^OH → Ag_n_ + (CH_3_)_2_CO + H^+^ (6)

The silver ions and the zero-valent silver atoms eventually aggregate to form larger AgNPs (Reactions (7)–(11)).

Ag^+^ + Ag^0^ → Ag_2_^+^ (7)

Ag_2_^+^ + Ag_2_^+^ → Ag_4_^2+^ (8)

Ag_i_^x+^ + Ag_m_^0^ → Ag_i+m_^x+^ (9)

Ag_j_^y+^ + Ag_k_^z+^ → Ag_j+k_^(y+z)+^ (10)

Ag_p_^0^ + Ag_q_^0^ → Ag_p+q_^0^ (11)

These association and coalescence reactions are influenced and limited by electrostatic and steric interactions due to the presence of charges and stabilizers. In particular, Mulvaney and Henglein showed by pulsed radiolysis experiments, using both Ag_2_SO_4_ and AgClO_4_ salts, that agglomeration processes were accelerated by sulfate ions compared to perchlorate ions [46]. It is also worth noticing that while SO_4_^2^^−^ and ClO_4_^−^ do not react with the generated radiolytic species, nitrate ions may slightly scavenge the hydrated electron (Reaction (12)):

NO_3_^−^ + e^−^_aq_ → NO_3_^2^^−^ (12)

The binding energy between two Ag atoms is greater than the Ag–PVA bond energy [47]. Additionally, any unreacted hydroxyl radicals can react with PVA to produce PVA^•^ radicals [48]. The resulting network formed by the cross-linking of PVA radicals can furthers prevent or stabilize the agglomeration of silver nanoparticles (Reaction (13)).

PVA^•^ + PVA^•^ → PVA–PVA (13)

**Figure 3:**
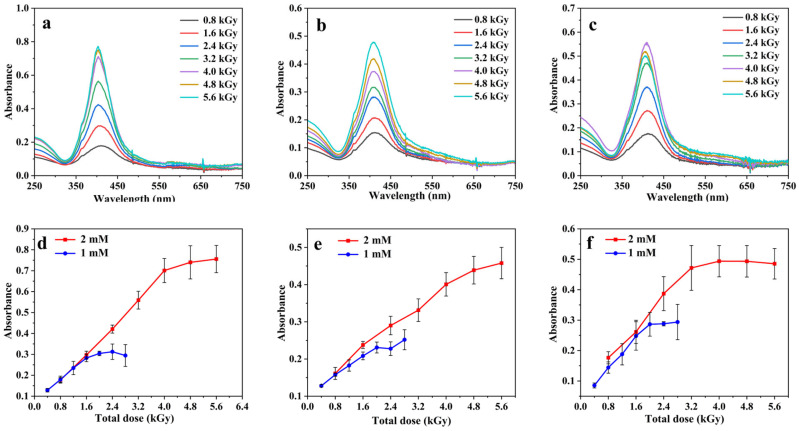
UV-vis absorption spectra recorded after dilution by a factor 40 of *γ*-irradiated aqueous solutions containing 0.1 M PVA, 0.1 M 2-propanol, and 2 mM [Ag+] from Ag_2_SO_4_ (**a**), AgNO_3_ (**b**), and AgClO_4_ (**c**) as a function of the absorbed dose. Maximum absorbance as a function of the dose for irradiated solutions containing 2 mM and 1 mM [Ag^+^] using Ag_2_SO_4_ (**d**), AgNO_3_ (**e**), and AgClO_4_ (**f**). The optical path length was 1.0 cm. The reference was water.

Figure 3 presents the absorption spectra recorded after dilution by a factor 40 of irradiated solutions containing 2 mM [Ag^+^] (from either Ag_2_SO_4_, AgNO_3,_ or AgClO_4_), 0.1 M PVA, and 0.1 M 2-propanol exposed to *γ*-rays as a function of the dose received by the samples, from 0.8 to 5.6 kGy (Figure 3a, Figure 3b and Figure 3c respectively). As the dose increases, the well-known plasmon absorption band of silver clusters around 400 nm appears and increases, indicating that the Ag^+^ cations are reduced, and that the metal atoms coalesce to form clusters stabilized by PVA. In the case of Ag_2_SO_4_ used as precursor, the spectra present a well-defined narrow band while for AgNO_3_ and AgClO_4_ the bands are less intense, broader, and show a tail and even a second absorption band on the red side, indicating less homogeneous nanoparticles. These results agree well with previous studies where the AgNPs synthesised using AgClO_4_ as precursor present a shoulder around 550–600 nm [21]. It is also worth noting that the wavelength of the absorption maximum is red-shifted when going from Ag_2_SO_4_ to AgClO_4_ and to AgNO_3_, suggesting the formation of larger particles. Figure 3d–f shows the maximum absorbance as a function of the dose when using [Ag^+^] at 1 mM and 2 mM for each of the assayed silver salt precursors. Over an absorbed dose of 2 and 4 kGy for [Ag^+^] equal to 1 and 2 mM, respectively, the spectrum remains nearly unchanged, and the maximum absorbance reaches a plateau indicating the total transformation of Ag^+^ ions into AgNPs. For AgNO_3_ as precursor, the plateau is reached at slightly higher doses compared to Ag_2_SO_4_ and AgClO_4_ due to Reaction (12) decreasing slightly the reduction yield.

**Figure 4:**
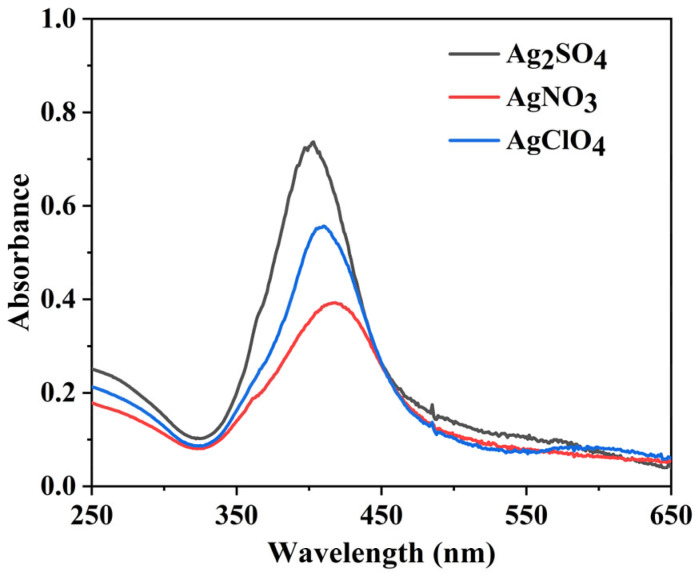
UV-vis absorption spectra of AgNPs synthesized using Ag_2_SO_4_, AgNO_3,_ and AgClO_4_ as precursors and at a total radiation dose of 4 kGy. The optical path length was 1.0 cm. The reference was water.

The difference in the absorbance spectra between the different salt precursors can be seen more clearly in Figure 4 for the same dose of 4 kGy and an initial [Ag^+^] of 2 mM. The maximum molar extinction coefficients per Ag^0^ can be estimated from the plateau and are found to be around 15,000, 10,000, and 9000 L·mol^−1^·cm^−1^ for Ag_2_SO_4_, AgClO_4,_ and AgNO_3_, respectively. Such values are in agreement with those already reported in the literature for AgNPs [21,49].

**Figure 5:**
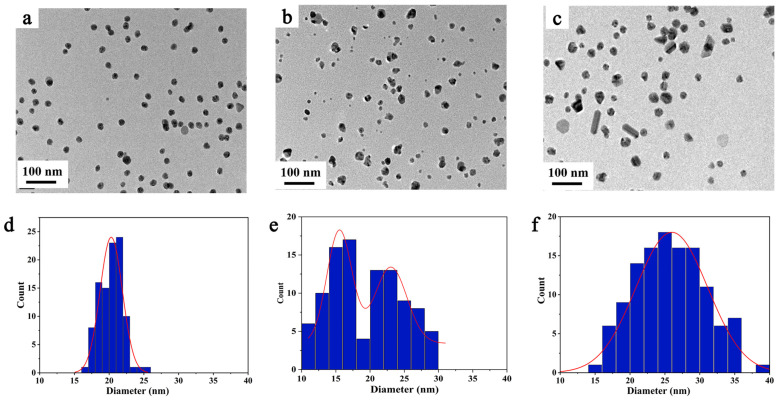
TEM images representing the morphology and size distribution of colloidal AgNPs prepared with Ag_2_SO_4_ (**a**,**d**), AgNO_3_ (**b**,**e**), and AgClO_4_ (**c**,**f**) as precursor, respectively. The size distribution was determined using Image J based on the analysis of at least 100 nanoparticles.

Following the characterization of the synthesized AgNPs using the three different Ag^+^ ions precursors, Figure 5 shows the TEM images as well as the size distribution histograms. As expected from the UV-vis absorption spectra, using Ag_2_SO_4_ as precursor produces quite homogeneous spherical AgNPs with a narrow size distribution (size = 20 ± 2 nm, Figure 5d). The mean hydrodynamic diameter of the AgNPs determined by DLS was found to be 26 ± 3 nm (see Table S1), in agreement with the previous value. The zeta potential of the AgNPs was close to −16 mV (Table S1) in aqueous solution containing 1 mM KCl at pH 7. This small negative value suggests that electrostatic repulsion may help in the stabilization of the suspensions in addition to the polymer coating steric stabilization [50].

In contrast, radiolysis of Ag^+^ solutions using AgNO_3_ or AgClO_4_ as precursors leads to AgNPs more dispersed in size (Figure 5e,f) and shape. With AgNO_3_, rather spherical AgNPs are obtained, but with a bimodal distribution in size ranging from less than 10 nm up to 30 nm. This can be explained by the fact that NO_3_^−^ reacts with solvated electrons leading to less reducing radicals, which can reduce silver only on the AgNP seeds. At the same time, some Ag^+^ ions are reduced on the previously formed AgNP seeds instead of coalescing into new ones. As a result, a bimodal size distribution is obtained. With AgClO_4_, most of the NPs are spherical, but other shapes (triangles and rods) are also observed. Such shapes account for the observed UV-visible absorption in the 550–650 nm spectral domain. Based on these results, the rest of the experiments were performed using Ag_2_SO_4_ as precursor with [Ag^+^] = 2 mM and a total dose of 4 kGy.

**Figure 6:**
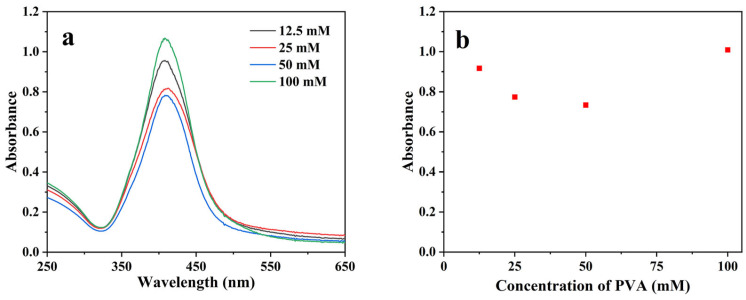
(**a**) UV-vis absorption spectra recorded after dilution by a factor 40 of *γ*-irradiated aqueous solutions containing 1 mM Ag_2_SO_4_, 0.1 M 2-propanol, and various concentrations of PVA (from 12.5 to 100 mM) after *γ*-irradiation with an absorbed dose of 4 kGy. (**b**) The maximum absorbance as a function of the PVA concentration. The optical path length was 1.0 cm. The reference was water.

Finally, the influence of the concentration of PVA used during the synthesis as stabilizer was evaluated. To do so, different concentrations of PVA (from 12.5 to 100 mM) were used for the synthesis while keeping unchanged the [Ag^+^] and the absorbed dose. As it can be seen in Figure 6, there are no significant differences in the absorption spectra and maxima as a function of the used PVA concentration. However, to ensure a good colloidal stability of the AgNPs, a PVA concentration of 100 mM was selected for the rest of the experiments.

### 3.2. AgNPs Stability Studies

**Figure 7:**
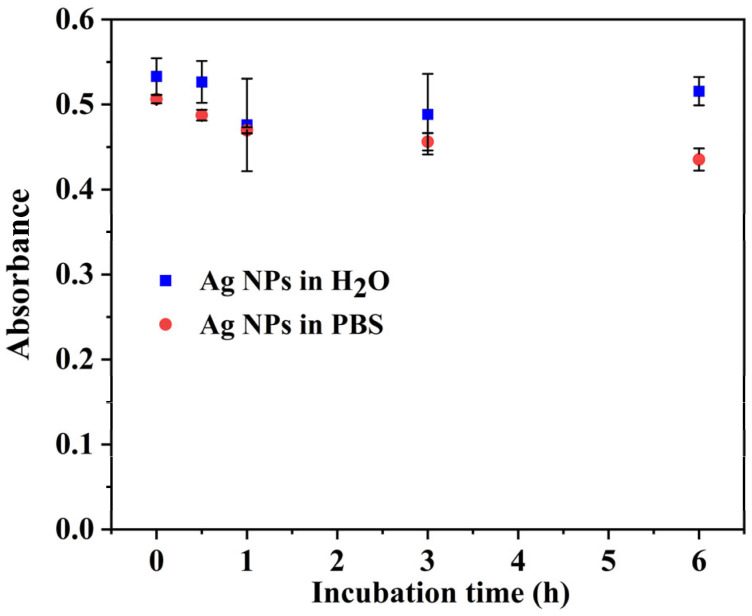
Stability of AgNPs derived from the Ag_2_SO_4_ precursor in deionized water and PBS medium in incubation time of 6 h. The optical path length was 1.0 cm. The reference was water.

In order to test the potential biological applications of the synthesized AgNPs, it was necessary to investigate their stability in different media, i.e., water and PBS (10 mM, pH = 7.4). So, 25 µL of the irradiated solution were diluted in 1 mL of either water or PBS. Figure 7 shows the maximum absorbance of the AgNP plasmon band as a function of time. There is no change in the absorbance intensity (nor in the absorbance spectrum) after 6 h in water and a diminution of less than 20% for the AgNPs diluted in PBS.

Based on the characterization results presented above, the AgNPs synthesized using 2 mM Ag_2_SO_4_, 100 mM PVA, and 0.1 M 2-propanol were selected for subsequent antibacterial testing.

### 3.3. Antibacterial Activity of AgNPs

**Figure 8:**
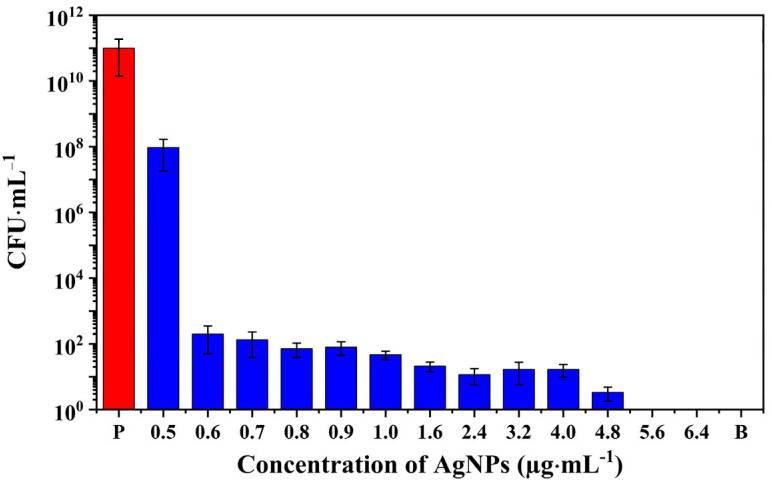
Antimicrobial activity against *S. aureus* of AgNPs synthesized by radiolysis using Ag_2_SO_4_, as precursor, as a function of their concentrations. P and B represent the positive control and the blank group, respectively.

The antibacterial activity of the AgNPs synthesized by γ-irradiation using Ag_2_SO_4_ as precursor was evaluated against the *S. aureus* strain ATCC 27217. The bactericidal ability of the as-prepared AgNPs was assessed using a standard broth microdilution method (Figure 8). Figure 8 demonstrates that the bacterial inoculum in the positive control group cultured overnight leads to a 5-order magnitude increase in the number of bacterial colonies. In turn, AgNP samples displayed antimicrobial activities as a function of their concentrations.

For instance, AgNPs, at a concentration of 0.5 μg·mL^−^^1^, failed to inhibit the *S. aureus* growth. However, AgNPs at a concentration of 0.6 μg·mL^−^^1^ significantly disrupted bacterial reproduction and demonstrated a powerful antibacterial ability. Thus, AgNPs inhibited the growth of *S. aureus* in a wide concentration range of 0.6–4.8 µg·mL^−^^1^, with only a small amount of bacterial growth observed in the agar plate (Figure S2). No bacterial growth was observed on agar plates when the tested bacteria were treated with nanoparticles at concentrations higher than 5.6 μg·mL^−^^1^ (Figure S2 and Figure 8).

The minimum inhibitory concentrations (MICs) and the minimum bactericidal concentrations (MBCs) are two useful quantitative indicators for the antimicrobial ability of NPs. MIC represents the lowest concentration of an antimicrobial that will inhibit the visible growth of a microorganism after overnight incubation, and MBC is defined as the lowest concentration of an antimicrobial that will prevent the growth of microorganism after subculture onto antibiotic-free media [51]. In this study, the MIC and MBC of the optimized AgNPs produced by radiolysis are 0.6 μg·mL^−^^1^ and 5.6 μg·mL^−^^1^, respectively.

Moreover, compared with results reported in the literature (Table S2), the antibacterial performance of the AgNPs produced by radiolysis is remarkable. Typically, except for a few examples, MIC ranges between 5 and 100 μg·mL^−^^1^, and MBC between 6 and 90 μg·mL^−^^1^. For instance, the formation of AgNPs by reduction in the presence of lignin was reported by Slavin et al. [13]. The resulting AgNPs, of around 20 nm, had spherical shapes and a good sterilization effect against MDR *S. aureus* (MIC and MBC values of about 10 μg·mL^−1^) and *S. aureus* ATCC 700,788 (MIC and MBC values of 5 μg·mL^−1^ and 10 μg·mL^−1^, respectively). Besides, Thammawithan et al. have also developed a series of AgNPs modulated by hydrogen peroxide under the reduction of tannic acid. They presented good antibacterial abilities against *S. aureus* ATCC 25,923 with MIC values in the range 8–100 μg·mL^−1^, and MBC values of 16–100 μg·mL^−1^ [52]. It is noteworthy to point out that other components used in the synthesis of AgNPs, such as PVA, lacked antibacterial potential against *S. aureus* (Figure S1). The unreacted silver mixture with ionic concentrations ranging from 0.6 to 3.2 μg·mL^−^^1^ displayed antibacterial activity against *S. aureus*. These results are in line with studies by Shi et al. [53], who demonstrated that both silver ions and AgNPs could cause significant damage to bacterial cells, resulting in the leakage of cytoplasmic contents and macromolecules, and eventually lead to cell death. Several mechanisms may account for the AgNP antibacterial abilities (Figure 1) [54,55,56,57]. Additionally, the release of bioactive silver ions may lead to the production of reactive oxygen species, which can further disrupt metabolic pathways, inhibit DNA replication, and affect the synthesis of related enzymes and proteins [55,56].

In conclusion, the optimized AgNPs have strong bactericidal effects against *S. aureus*, compared to other AgNPs obtained from different synthesis methods (Table S2). Radiolytic synthesis has many advantages compared to chemical reduction. For example: (i) the reducing agents are induced from solvent radiolysis (no chemical reducing agents are necessary); (ii) solvated electrons are very powerful reducing agents (they can reduce metal ions, which are difficult to reduce at room temperature by chemical methods); (iii) the reduction is very homogeneous as well as the nucleation, leading to nanoparticles with very narrow size distributions; (iv) the synthesis can be carried out in confined media [17,20,21].

In addition, radiolysis sterilizes the AgNP suspensions, which is beneficial for biological applications. Our results show that the resulting antibacterial properties are among the highest reported in the literature as compared to other synthesis methods (Table S2). To explore the full potential of our AgNPs obtained by radiolysis, further in-depth investigations of their toxicity and scale-up production will be carried out in the near future.

## 4. Conclusions

This study has explored, for the first time, the influence of silver salt precursors (Ag_2_SO_4_, AgNO_3_, and AgClO_4_) on the optical properties, particle size, and morphology of colloidal AgNPs synthesized via *γ*-irradiation. The optical absorption of the AgNP solutions (using various silver salts as precursors) increases with the irradiation dose until reaching a plateau at 4 kGy for 2 mM and 2 kGy for 1 mM initial concentration in Ag^+^, respectively. Of note, AgNPs obtained from Ag_2_SO_4_ at a concentration of 2 mM exhibit the highest absorbance, with a molar extinction coefficient of approximately 15,000 M^−1^·cm^−1^ and a narrow UV-visible absorption band. Furthermore, AgNPs synthesized from Ag_2_SO_4_ (2 mM) display a spherical morphology with high homogeneity and a narrow size distribution of 20 ± 2 nm, in contrast to those produced from AgNO_3_ and AgClO_4_. Interestingly, the concentration of PVA (in the range 12.5 to 100 mM) in the colloidal AgNPs does not significantly impact the optical properties of the particles.

Moreover, the optimized AgNPs obtained from Ag_2_SO_4_ at a concentration of 2 mM are stable upon storage in water and PBS, while being effective in inhibiting the growth of *S. aureus* at a concentration of 0.6 μg·mL^−1^ and completely eradicating it at a concentration of 5.6 μg·mL^−1^. The AgNPs prepared by radiolysis are sterile and might also have potential in eradicating other pathogens. Their outstanding bactericidal ability against *S. aureus,* compared to various AgNPs prepared from conventional methods and other different strategies, opens new avenues for further applications in medicine, cosmetics, and the food industry, as well as in the manufacture of antibacterial surfaces or other devices.

## Data Availability

Data are available upon request.

## Supplementary Materials

The following supporting information can be downloaded at: https://www.mdpi.com/article/10.3390/pharmaceutics15071787/s1, Figure S1: Antimicrobial performance of silver reaction mixture at different ionic concentrations from 0.6 to 3.2 μg·mL^−1^, combination of PVA (50 mM) and 2-propanol (100 mM), positive control, and blank control group against *S. aureus* (P represents the positive control group. C represents the combination of PVA and 2-propanol. B represents the blank group); Figure S2: Antibacterial activity of optimized AgNPs prepared using the Ag_2_SO_4_ precursor, positive control, blank control, silver mixture before synthesis, and combination of PVA and 2-propanol. The incubated solutions were serially diluted and deposited on an agar plate before incubation at 37 °C; Table S1: AgNPs mean hydrodynamic diameters and zeta potential after pH adjustment at 7 and 30 times dilution in a 1 mM KCl solution. The measurements were made in triplicate with optimized AgNPs (AgNP-1, AgNP-2, and AgNP-3) obtained starting from the Ag_2_SO_4_ precursor with an ionic concentration of 2 mM; Table S2: Antibacterial properties (based on MICs and MBCs) for different AgNPs. References [13,14,19,32,35,52,58,59,60,61,62,63,64,65,66] are cited in the supplementary materials.
