# Supplementary material for: Colloidal Silver Nanoparticles Obtained via Radiolysis: Synthesis Optimization and Antibacterial Properties
Source: Pharmaceutics. 2023 Jun 21;15(7):1787. doi: 10.3390/pharmaceutics15071787 (PMC10383763; doi:10.3390/pharmaceutics15071787)
Supplement: Supplementary file 1 [file pharmaceutics-15-01787-s001.zip › pharmaceutics-2442114-supplementary.pdf]

Supporting Information

Colloidal Silver Nanoparticles Obtained via Radiolysis: Synthesis Optimization and Antibacterial Properties

Mario Menéndez Miranda [1,†], Wenbo Liu [1,†], Jesus Alfredo Godinez-Leon [1], Aisara Amanova [2], Ludivine Houel-Renault [1], Isabelle Lampre [2], Hynd Remita [2,*] and Ruxandra Gref [1,*]

1 Institut de Sciences Moléculaires d'Orsay, CNRS-UMR 8214, Université Paris-Saclay, 91400 Orsay, France; mmiranda@serida.org (M.M.M.); wenbo.liu@universite-paris-saclay.fr (W.L.); jesus.godinez@universite-paris-saclay.fr (J.A.G.-L.); ludivine.houel-renault@universite-paris-saclay.fr (L.H.-R.)

2 Institut de Chimie Physique, CNRS-UMR 8000, Université Paris-Saclay, 91405 Orsay, France; aisara.amanova@universite-paris-saclay.fr (A.A.); isabelle.lampre@universite-paris-saclay.fr (I.L.)

* Correspondence: hynd.remita@universite-paris-saclay.fr (H.R.); ruxandra.gref@universite-paris-saclay.fr (R.G.)

† These authors contributed equally to this work.

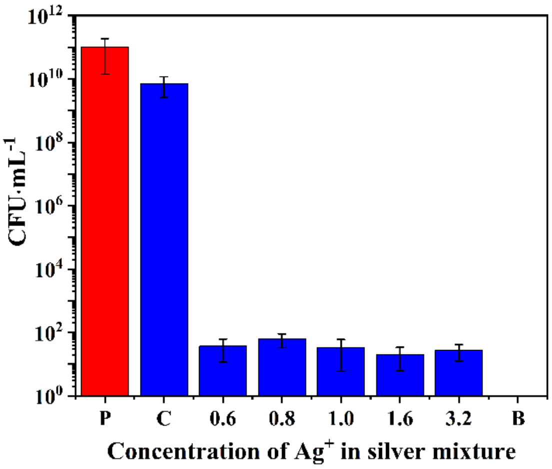

Figure S1. Antimicrobial performance of silver reaction mixture at different ionic concentrations from 0.6 to 3.2 μg·mL^{-1}, combination of PVA (50 mM) and 2-propanol (100 mM), positive control, and blank control group against *S.aureus* (P represents the positive control group. C represents the combination of PVA and 2-propanol. B represents the blank group).

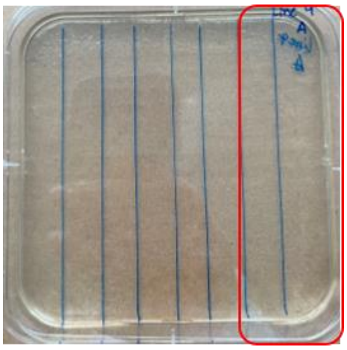

S.aureus + AgNPs with 6.4 μg·mL^{-1} / LB + AgNPs with 6.4 μg·mL^{-1} (Red circle)

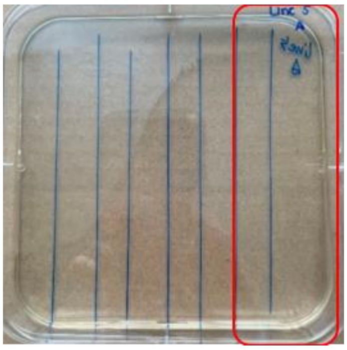

S.aureus + AgNPs with 5.6 μg·mL^{-1} / LB + AgNPs with 5.6 μg·mL^{-1} (Red circle)

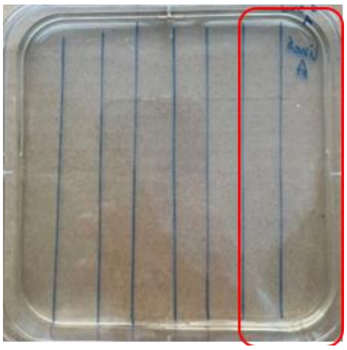

S.aureus + AgNPs with 4.8 μg·mL^{-1} / LB + AgNPs with 4.8 μg·mL^{-1} (Red circle)

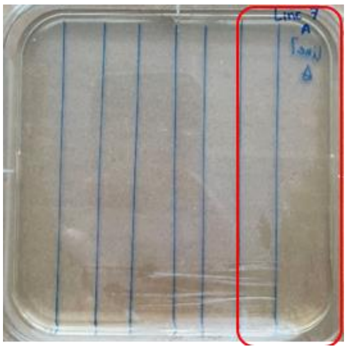

S.aureus + AgNPs with 4.0 μg·mL^{-1} / LB + AgNPs 4.0 μg·mL^{-1} (Red circle)

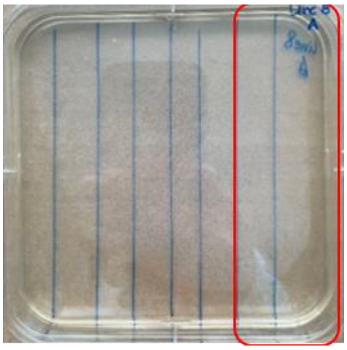

S.aureus + AgNPs with 3.2 μg·mL^{-1} / LB + AgNPs 3.2 μg·mL^{-1} (Red circle)

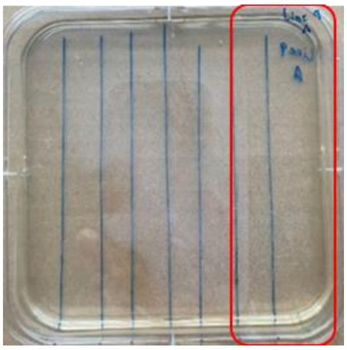

S.aureus + AgNPs with 2.4 μg·mL^{-1} / LB + AgNPs 2.4 μg·mL^{-1} (Red circle)

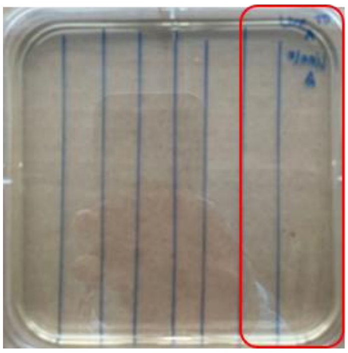

S.aureus + AgNPs with 1.6 μg·mL^{-1} / LB + AgNPs 1.6 μg·mL^{-1} (Red circle)

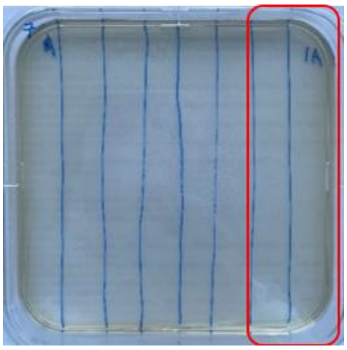

S.aureus + AgNPs with 1.0 μg·mL^{-1} / LB + AgNPs 1.0 μg·mL^{-1} (Red circle)

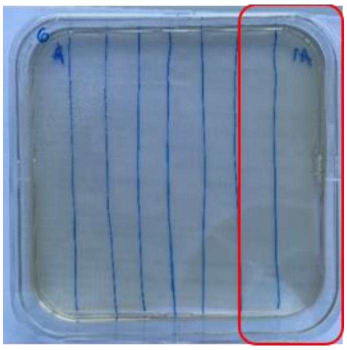

S.aureus + AgNPs with 0.9 μg·mL^{-1} / LB + AgNPs 0.9 μg·mL^{-1} (Red circle)

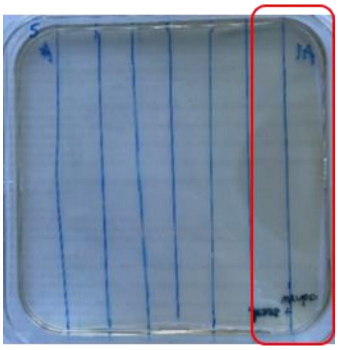

S.aureus + AgNPs with 0.8 μg·mL^{-1} /

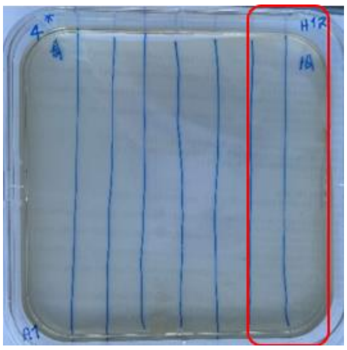

S.aureus + AgNPs with 0.7 μg·mL^{-1} /

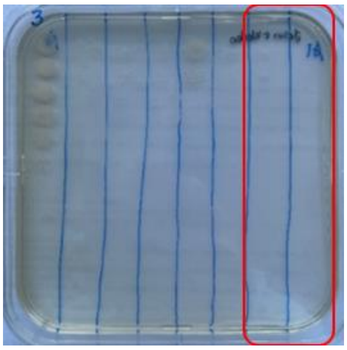

S.aureus + AgNPs with 0.6 μg·mL^{-1} /

LB + AgNPs 0.8 µg·mL^{-1} (Red circle) LB + AgNPs 0.7 µg·mL^{-1} (Red circle) LB + AgNPs 0.6 µg·mL^{-1} (Red circle)

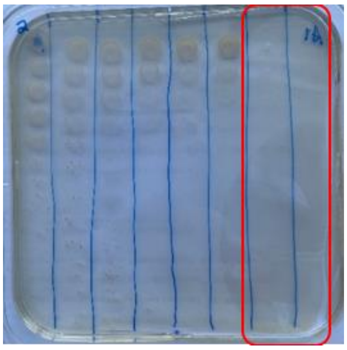

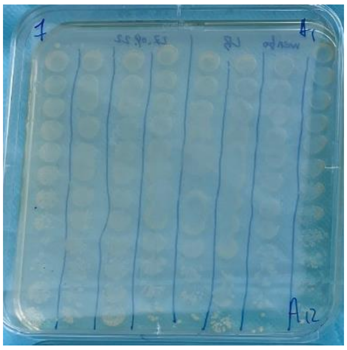

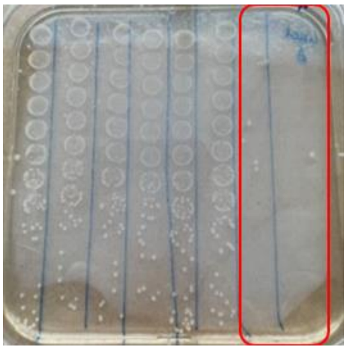

S.aureus + AgNPs with 0.5 µg·mL^{-1} / LB broth + AgNPs 0.5 µg·mL^{-1} (Red circle)

Positive control

S.aureus + combination of PVA + 2-proponal / LB + combination of PVA + 2-proponal) (Red circle)

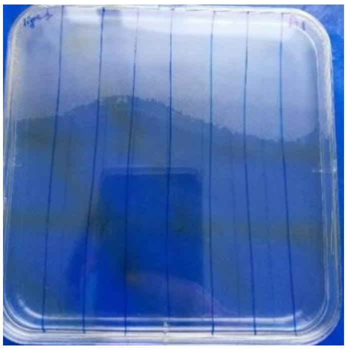

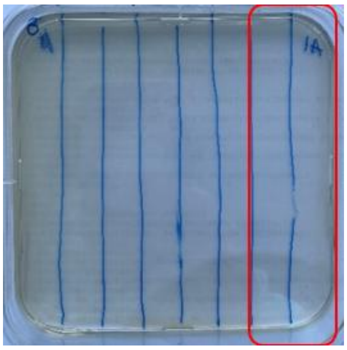

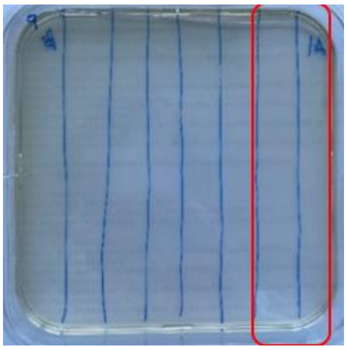

Blank control

S.aureus + Ag mixture with 0.6 µg·mL^{-1} / LB + Ag mixture with 0.6 µg·mL^{-1} (Red circle)

S.aureus + Ag mixture with 0.8 µg·mL^{-1} / LB + Ag mixture with 0.8 µg·mL^{-1} (Red circle)

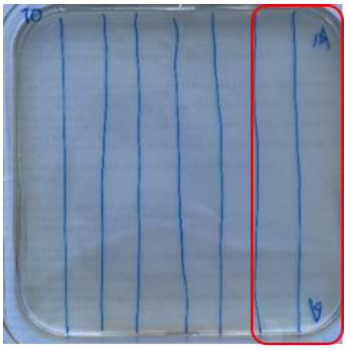

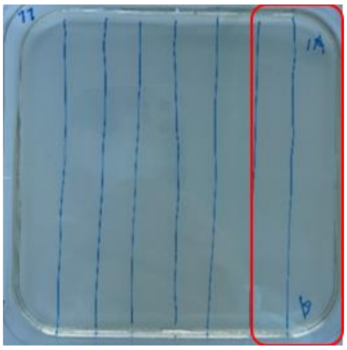

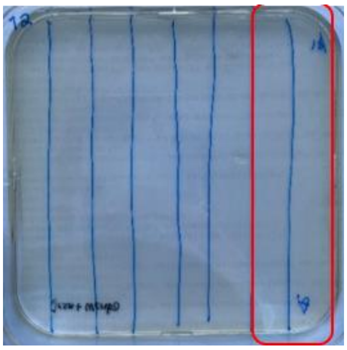

S.aureus + Ag mixture with 1.0 µg·mL^{-1} / LB + Ag mixture with 1.0 µg·mL^{-1} (Red circle)

S.aureus + Ag mixture with 1.6 µg·mL^{-1} / LB + Ag mixture with 1.6 µg·mL^{-1} (Red circle)

S.aureus + Ag mixture with 3.2 µg·mL^{-1} / LB + Ag mixture with 3.2 µg·mL^{-1} (Red circle)

Figure S2. Antibacterial activity of optimized AgNPs prepared using the Ag_2SO_4 precursor, positive control, blank control, silver mixture before synthesis, and combination of PVA and 2-propanol. The incubated solutions were serially diluted and deposited on the agar plate before incubation at 37 °C.

Table S1. AgNPs mean hydrodynamic diameters and zeta potential after pH adjustment at 7 and 30 times dilution in a 1 mM KCl solution. The measurements were made in triplicate with optimized AgNPs (AgNP-1, AgNP-2 and AgNP-3) obtained starting from the Ag_2SO_4 precursor with an ionic concentration of 2 mM.

Sample Name	Z-Ave (d. nm)	Zeta Potential (mV)
AgNP-1	26 ± 2	–15 ± 3
AgNP-2	27 ± 3	–16 ± 3
AgNP-3	25 ± 2	–16 ± 3

Table S2. Antibacterial properties (based on MICs and MBCs) for different AgNPs.

Silver form	Method	Size data	Bacterial strain	MIC	MBC	Ref.
Ag NPs-Myramistin	Chemical reduction	8.5 nm	*S.aureus* INA 00761	5 $\mu g \cdot mL^{-1}$	-	[1]
Lignin-Ag NPs	Chemical reduction	~20 nm	*S.aureus* MDR	10 $\mu g \cdot mL^{-1}$	10 $\mu g \cdot mL^{-1}$	[2]
			S.aureus ATCC700788	5 $\mu g \cdot mL^{-1}$	10 $\mu g \cdot mL^{-1}$	
PSS-Ag NPs	Chemical reduction	5 nm	*S.aureus* ATCC29213	1.14 $\mu g \cdot mL^{-1}$	-	[3]
Dendrimer-encapsulated-Ag NPs	Chemical reduction	3.33 nm	*S.aureus* USA300	128 $\mu g \cdot mL^{-1}$	-	[4]
Carboxy methyl cellulose-Ag NPs	Chemical reduction	5–15 nm	*S.aureus* ATCC43300	60 $\mu g \cdot mL^{-1}$	60 $\mu g \cdot mL^{-1}$	[5]
AgNPs-Tannic acid	Chemical reduction	4.69 ± 1.56 nm	*S.aureus* ATCC 25923	8–16 $\mu g \cdot mL^{-1}$	16-32 $\mu g \cdot mL^{-1}$	[6]
Zeolite containing silver and zinc	Chemical reduction	ND	*S.aureus* ATCC 25923	-	39 $\mu g \cdot mL^{-1}$	[7]
Ag NPs-Cotyledon orbiculate	Biosynthesis reduction	106–137 nm ± 2	*S.aureus* ATCC 25923	20 $\mu g \cdot mL^{-1}$	40 $\mu g \cdot mL^{-1}$	[8]
Ag NPs-A. esculentus flower extract	Biosynthesis reduction	5.52–24.65 nm; average	*S.aureus* ATCC29213	85 $\mu g \cdot mL^{-1}$	90 $\mu g \cdot mL^{-1}$	[9]

		18.24				
Ag NPs-lyophilized hydroalcoholic extract of S. Cumini seeds	Biosynthesis reduction	36.25–77.01 nm	*S.aureus* ATCC25923	125 μg·mL^{-1}	-	[10]
Ag NPs-Artemisia haussknechtii leaf aqueous extract	Biosynthesis reduction	10.69 nm	*S.aureus* ATCC 43300	10 μg·mL^{-1}	60 μg·mL^{-1}	[11]
AgNPs-Sucrose	Saccharide-based reduction	25 ± 5 nm	*S.aureus*	0.057 μg·mL^{-1}	0.23 μg·mL^{-1}	[12]
AgNPs-Soluble starch	Saccharide-based reduction	25 ± 5 nm	*S.aureus*	0.34 μg/mL	1.62 μg/mL	
Ag NPs-PVA(72000)	γ-Radiation reduction	~28 nm	*S.aureus*	-	-	[13]
AgNPs-Chitosan-derived CQDs	γ-Radiation reduction	25 nm	*S.aureus*	100 μg·mL^{-1}	-	[14]
AgNPs-PVA(1700-1800)	γ-Radiation reduction	31.2–50.8 nm	*S.aureus* ATCC29213	-	-	[15]
AgNPs-PVA(30000 ~ 70000)	γ-Radiation reduction	20 nm	*S.aureus* ATCC 27217	0.6 μg·mL^{-1}	5.6 μg·mL^{-1}	**Optimized NPs in this work**

References

1. Vertelov, G.K.; Krutyakov, Y.A.; Efremenkova, O.V.; Olenin, A.Y.; Lisichkin, G.V. A Versatile Synthesis of Highly Bactericidal Myramistin® Stabilized Silver Nanoparticles. *Nanotechnology.* **2008**, *19*, 355707.
2. Slavin, Y.N.; Ivanova, K.; Hoyo, J.; Perelshtein, I.; Owen, G.; Haegert, A.; Lin, Y.-Y.; LeBihan, S.; Gedanken, A.; Häfeli, U.O.; et al. Novel Lignin-Capped Silver Nanoparticles against Multidrug-Resistant Bacteria. *ACS Appl. Mater. Interfaces.* **2021**, *13*, 22098–22109.
3. Bartel, M.; Markowska, K.; Strawski, M.; Wolska, K.; Mazur, M. Silver-Decorated Gel-Shell Nanobeads: Physicochemical Characterization and Evaluation of Antibacterial Properties. *Beilstein J. Nanotechnol.* **2020**, *11*, 620–630.
4. Dai, T.; Wang, C.; Wang, Y.; Xu, W.; Hu, J.; Cheng, Y. A Nanocomposite Hydrogel with Potent and Broad-Spectrum Antibacterial Activity. *ACS Appl. Mater. Interfaces.* **2018**, *10*, 15163–15173.
5. Ambi, A.; Parikh, N.; Vera, C.; Burns, K.; Montano, N.; Sciorra, L.; Epstein, J.; Zeng, D.; Traba, C. Anti-Infection Silver

Nanoparticle Immobilized Biomaterials Facilitated by Argon Plasma Grafting Technology. *Biofouling.* **2018**, *34*, 273–286.

6. Thammawithan, S.; Siritongsuk, P.; Nasompag, S.; Daduang, S.; Klaynongsruang, S.; Prapasarakul, N.; Patramanon, R. A Biological Study of Anisotropic Silver Nanoparticles and Their Antimicrobial Application for Topical Use. *Vet. Sci.* **2021**, *8*, 177.
7. Cowan, M.M.; Abshire, K.Z.; Houk, S.L.; Evans, S.M. Antimicrobial Efficacy of a Silver-Zeolite Matrix Coating on Stainless Steel. *J. Ind. Microbiol. Biotechnol.* **2003**, *30*, 102–106.
8. Tyavambiza, C.; Elbagory, A.M.; Madiehe, A.M.; Meyer, M.; Meyer, S. The Antimicrobial and Anti-Inflammatory Effects of Silver Nanoparticles Synthesised from Cotyledon Orbiculata Aqueous Extract. *Nanomaterials.* **2021**, *11*, 1343.
9. Devanesan, S.; AlSalhi, M.S. Green Synthesis of Silver Nanoparticles Using the Flower Extract of Abelmoschus Esculentus for Cytotoxicity and Antimicrobial Studies. *Int. J. Nanomedicine.* **2021**, *16*, 3343.
10. de Carvalho Bernardo, W.L.; Boriollo, M.F.G.; Tonon, C.C.; da Silva, J.J.; Cruz, F.M.; Martins, A.L.; Höfling, J.F.; Spolidorio, D.M.P. Antimicrobial Effects of Silver Nanoparticles and Extracts of Syzygium Cumini Flowers and Seeds: Periodontal, Cariogenic and Opportunistic Pathogens. *Arch. Oral Biol.* **2021**, *125*, 105101.
11. Alavi, M.; Karimi, N. Characterization, Antibacterial, Total Antioxidant, Scavenging, Reducing Power and Ion Chelating Activities of Green Synthesized Silver, Copper and Titanium Dioxide Nanoparticles Using Artemisia Haussknechtii Leaf Extract. *Artif. Cells Nanomedicine Biotechnol.* **2018**, *46*, 2066–2081.
12. Valodkar, M.; Bhadoria, A.; Pohnerkar, J.; Mohan, M.; Thakore, S. Morphology and Antibacterial Activity of Carbohydrate-Stabilized Silver Nanoparticles. *Carbohydr. Res.* **2010**, *345*, 1767–1773.
13. Uttayarat, P.; Eamsiri, J.; Tangthong, T.; Suwanmala, P. Radiolytic Synthesis of Colloidal Silver Nanoparticles for Antibacterial Wound Dressings. *Adv. Mater. Sci. Eng.* **2015**, 2015, 376082.
14. Thu, H.T.; Anh, L.T.; Phuc, L.H.; Vinh, L.K.; Tung, N.T.; Phuong, P.H. Green Preparation of Carbon Quantum Dots and Its Silver Nanoparticles Composite against Carbapenem-Resistant Acinetobacter Baumannii. *Appl. Nanosci.* **2023,** *13, 4109-4118*.
15. Khozemy, E.E.; Nasef, S.M.; Mahmoud, G.A. Synthesis and Characterization of Antimicrobial Nanocomposite Hydrogel Based on Wheat Flour and Poly (Vinyl Alcohol) Using γ-Irradiation. *Adv. Polym. Technol.* **2018**, *37*, 3252–3261.
